# Differences in the association of oral health knowledge, attitudes, and practices with frailty among community-dwelling older people in China

**DOI:** 10.1186/s12903-023-03477-y

**Published:** 2023-10-24

**Authors:** Chenglin Cao, Shengdong Liao, Wenwen Cao, Ying Guo, Zixuan Hong, Bohua Ren, Zhi Hu, Zhongliang Bai

**Affiliations:** 1 Department of Health Services Management, School of Health Services Management, Anhui Medical University, Hefei, Anhui 230032 China; 2 Academic Affairs Office, Anhui Medical University, Hefei, Anhui 230032 China; 3 Department of Epidemiology and Biostatistics, School of Public Health, Guangdong Medical University, Dongguan, Guangdong 523808 China; 4 Faculty of Education, Health and Wellbeing, University of Wolverhampton, Wolverhampton, WV1 1QU UK; 5 Key Laboratory of Public Health Social Governance, Philosophy and Social Sciences of Anhui Province, Hefei, Anhui 230032 China

**Keywords:** Oral health knowledge, Oral health attitudes, Oral health practices, Frailty, Older people

## Abstract

**Background:**

Oral health and frailty are significantly related and should be well examined, especially in late life. Few studies have explored the relationship of oral health knowledge, attitudes, and practices with frailty and examined sociodemographic variations in this association. This study aimed to examine the association between oral health knowledge, attitudes, practices and frailty, with a special focus on comparing differences in their association among the Chinese community-dwelling older population.

**Methods:**

This study included 4218 community-dwelling older adults (aged ≥ 60 years) who participated in a cross-sectional survey. Sociodemographic characteristics, oral health knowledge, attitudes, practices, and frail status (non-frailty, pre-frailty, and frailty) were collected with a face-to-face questionnaire-based interview. Multivariate logistic regression models were used to evaluate the association of oral health knowledge, attitudes, and practices with frailty.

**Results:**

Of the 4218 participants, 36.2% (n = 1527) and 18.8% (n = 792) were classified as pre-frailty and frailty. Age, gender and educational attainments differences existed in the association of oral health knowledge with frailty. Urban-rural differences in the association of oral health knowledge and practices with frailty were also found. Specifically, oral health knowledge was significantly related to frailty only among participants aged 70–79 years (adjusted odds ratio [95% confidence interval]) (1.08 [1.02–1.15]), females (1.05 [1.00–1.10]), rural residents (1.06 [1.01–1.12]), and those who were primary school and lower education (1.06 [1.01–1.11]), whereas oral health practices were related to frailty only among urban participants (0.96 [0.92–1.00]).

**Conclusion:**

This study confirmed the different associations of oral health knowledge and practices with frailty among community-dwelling older people in China. Further research is needed to better understand the abovementioned differences and public health strategies are required to improve oral health literacy and thereby contain the development of frailty in later life.

**Supplementary Information:**

The online version contains supplementary material available at 10.1186/s12903-023-03477-y.

## Introduction

An increasing proportion of the aging population has exacerbated the incidence of frailty today [1, 2]. Frailty is a complex aging-related condition, resulting in a decreased physiological capacity of organ systems and enhanced susceptibility to stressors [3]. As an emerging global health problem challenge, frailty puts a great burden and challenge not only on older individuals but also on their families and society [4, 5]. Therefore, the exploration of frailty-associated factors has great significance for academic and practical research.

World Health Organisation (WHO) pointed out that oral health remains a significant, but overlooked, component of healthy aging and well-being in older people [6, 7]. Oral health literacy (OHL), which comprises oral health knowledge, attitudes, and practices, represents an individual’s motivation and ability to access, understand, and use information through cognitive and social skills to promote and maintain oral health [8]. The importance of oral health literacy in the oral care practice and oral health status has been recognized previously [9, 10]. Besides, much attention has been paid to the impact of oral health-related indicators on frailty in later life [11]. For example, oral health practices, especially infrequent brushing or dental service usage, were confirmed related to frailty in older adults [12]. In addition, decreased tooth numbers and increased demand for dentures were linked to a greater risk of frailty in later life [13]. Also, a systematic review revealed that self-reported oral pain or masticatory disorders are significantly associated with frailty [14].

The presence of oral health-related indicators and frailty has been confirmed to vary in terms of various demographic factors [15–17]. Previous studies have found that advanced age, females, residing in rural, and less educational attainment had a higher prevalence of frailty in later life [18–20]. Besides, compared with males, females were more likely to have more positive attitudes towards dental healthcare services and higher oral health attitudes, better self-care practices, and higher oral health literacy [21]. Furthermore, self-care-related attitudes and behaviours are influenced by the educational level among older males [22]. In a recent study, barriers to accessing remote services and health facilities were linked to the generally poor oral health status of rural older people, compared to their urban counterparts [23]. Therefore, differences in these factors should be examined when oral health literacy-related variables are linked to frailty.

However, to date, few studies have explored the influence of sociodemographic disparities on the association of oral health knowledge, attitudes, practices with frailty in older adults. Given this, this paper aimed to investigate the association of oral health knowledge, attitudes, and practices with frailty, with a special focus on examining the differences in age, gender, residence, and educational attainment. The findings would contribute to providing evidence on the future supportive strategies to improve frailty in China community-dwelling older people through the development of personalised oral policies, tailored to age, gender, residence, and educational attainment separately.

## Materials and methods

### Sampling and recruitment

In November–December 2020, a multi-stage stratified-cluster random sampling-based cross-sectional survey was conducted at Jinshan of Shanghai; Huzhou of Zhejiang Province; Changzhou of Jiangsu Province; and Huainan of Anhui Province. We categorised the participants into urban and rural based on their household registration and residence information. In other words, those whose household registration was in urban areas and who live in urban areas were included in the urban population, and while were included in the rural population. With the support and coordination of local community workers, trained graduate students used structured questionnaires to conduct face-to-face interviews with the participants. The eligibility criteria for this study were: age ≥ 60 years, no obvious communicative or cognitive impairments, and willingness to participate in the study.

After excluding invalid questionnaires, 4218 valid questionnaires were included in this study. Prior to any data collection, each participant or their legally authorised representative provided written informed consent for participation in this study. For more information on the study design and data collection process for this paper, please refer to our previously published articles [24, 25].

## Measures

### Independent variables

On the basis of the 4th Chinese National Oral Health Epidemiological Survey [26], a structured questionnaire was developed and implemented in accordance with the procedures described in Oral Health Surveys Basic Methods 5th Edition [27]. Our study elicited data on oral health knowledge, attitudes, and practices among older people. (Full content has been provided in Supplementary Material S1).

For the 8 questionnaire items on oral health knowledge, responses were coded as correct, unknown, or incorrect (2, 1, or 0 points, respectively; total score: 0–16). Oral health attitudes were assessed from 6 items (total score: 0–12; negative attitude, 0 points; gradual positive attitude: 1–2 points), with a higher score indicating a more positive attitude towards oral health. For each of the 12 items on oral health practices (per item, maximum score: 3 points; minimum score: 0 points; total score: 0–36), higher scores were associated with less frequent adverse behaviours and more regulated oral health practices.

### Outcome variables

In this study, frailty was assessed with a four-dimensional (physical, psychological, social, and environmental), 23-item measurement tool. First, we calculated the score for each item and divided it by 23 separately to obtain the frailty score, classified as follows: <0.12 as non-frailty, 0.12–0.24 as pre-frailty, and ≥ 0.25 as frailty [25, 28]. The Cronbach’s alpha of 0.771 proved that the scale had good internal consistency. Detailed content of this measurement has been provided in Supplementary Material S1.

### Co-variables

Based on the findings of a literature review [20, 29–31], data on other variables, such as age (years), body mass index (BMI, kg/m^2^), gender (male or female), educational attainment (primary school and lower, junior school, or high school and above), residence (urban or rural), living status (living alone, living with others, etc.), and marital status, were also included. Moreover, data on smoking and drinking status, sources of income (salary, pension, family providing, subsidy, and others), depressive status, and functional ability (robust or limited) were also obtained.

### Data analysis

Based on the presence of frail status (non-frailty, pre-frailty, and frailty), the participants were divided into three groups. The participant demographics were presented through descriptive statistics. Logistic regression models were established to identify differences in the correlation of oral health knowledge, attitudes, and practices with frail status.

Multiple logistic regression analyses were conducted accordingly to determine differences, concerning age, gender, residence, and educational attainment, in the unadjusted and adjusted relationships between oral health knowledge, attitudes, practices and frailty. With reference to literatures [31, 32], we adjusted for potential covariates to obtain multiple logistic regression models. The adjusted odds ratio (AOR) and the corresponding 95% confidence interval (95% CI) were used to express the results of these models. All analyses in this study were conducted in SPSS 23.0.

## Results

### Participant characteristics

The characteristics of the participants, stratified by frail status as non-frailty (n = 1899/4218, 45.0%), pre-frailty (1527/4218, 36.2%), and frailty (792/4218, 18.8%), are shown in Table 1: 42.2% (n = 1779/4218) of participants were in the 60–69 age group, and the majority of respondents were female (n = 2734, 65.8%), urban residents (n = 2316, 54.9%), and lived with their family (n = 3650, 86.5%); 61.0% of older adults had primary or lower education (n = 2571/4218) and 78.8% (n = 3325/4218) were married/cohabiting. With regard to health practices, the majority of participants (n = 3332, 79.0%) had never smoked or consumed alcohol (n = 3386, 80.3%). More than half of the participants had no depression (n = 2355, 55.8%), but 45.6% (n = 1923/4218) had limited functional ability.

**Table 1:** Demographic, socioeconomic and oral health characteristics of the study sample of older adults stratified by frail status (N = 4218) [N (%)]

Variables	Categories	Frail status			Total N = 4218
		Non-frailty (n = 1899)	Pre-frailty (n = 1527)	Frailty (n = 792)	
Age (years)	60–69	914 (48.1)	595 (39.0)	270 (34.1)	1779 (42.2)
	70–79	747 (39.3)	651 (42.6)	340 (42.9)	1738 (41.2)
	≧ 80	238 (12.5)	281 (18.4)	182 (23.0)	701 (16.6)
Gender	Male	703 (37.0)	555 (36.3)	226 (28.5)	1484 (35.2)
	Female	1196 (63.0)	972 (63.7)	566 (71.5)	2734 (64.8)
BMI (kg/m^2^)	≦ 18.5	73 (3.8)	77 (5.0)	60 (7.6)	210 (5.0)
	18.5–22.9	637 (33.5)	474 (31.0)	272 (34.3)	1383 (32.8)
	23–27.4	940 (49.5)	766 (50.2)	353 (44.6)	2059 (48.8)
	≧ 27.5	249 (13.1)	210 (13.8)	107 (13.5)	566 (13.4)
Residence	Urban	1147 (60.4)	809 (53.0)	360 (45.5)	2316 (54.9)
	Rural	752 (39.6)	718 (47.0)	432 (54.5)	1902 (45.1)
Living status	Living alone	143 (7.5)	229 (15.0)	196 (24.7)	568 (13.5)
	Living with others	1756 (92.5)	1298 (85.0)	596 (75.3)	3650 (86.5)
Marital status	Married/cohabited	1589 (83.7)	1203 (78.8)	533 (67.3)	3325 (78.8)
	Single	310 (16.3)	324 (21.2)	259 (32.7)	893 (21.2)
Educational attainment	Primary school and below	1058 (55.7)	938 (61.4)	575 (72.6)	2571 (61.0)
	Junior school	467 (24.6)	346 (22.7)	124 (15.7)	937 (22.2)
	High school and above	374 (19.7)	243 (15.9)	93 (11.7)	710 (16.8)
Smoking status	Smoking-quitter	116 (6.1)	124 (8.1)	67 (8.5)	307 (7.3)
	Smoker	287 (15.1)	219 (14.3)	73 (9.2)	579 (13.7)
	Non-smoker	1496 (78.8)	1184 (77.5)	652 (82.3)	3332 (79.0)
Drinking status	Drinking-quitter	66 (3.5)	74 (4.8)	50 (6.3)	190 (4.5)
	Drinker	324 (17.1)	256 (16.8)	62 (7.8)	642 (15.2)
	Non-drinker	1509 (79.5)	1197 (78.4)	680 (85.9)	3386 (80.3)
Income source	Salary	123 (6.5)	173 (11.3)	94 (11.9)	390 (9.2)
	Pension	1186 (62.5)	806 (52.8)	352 (44.4)	2344 (55.6)
	Family providing	310 (16.3)	319 (20.9)	215 (27.1)	844 (20.0)
	Subsidy	190 (10.0)	169 (11.1)	101 (12.8)	460 (10.9)
	Others	90 (4.7)	60 (3.9)	30 (3.8)	180 (4.3)
Depressive status	No depression	1165 (61.3)	762 (49.9)	428 (54.0)	2355 (55.8)
	Minimal to mild depression	731 (38.5)	747 (48.9)	326 (41.2)	1804 (42.8)
	Depression	3 (0.2)	18 (1.2)	38 (4.8)	59 (1.4)
Functional ability	Robust	1240 (65.3)	804 (52.7)	251 (31.7)	2295 (54.4)
	Limited	659 (34.7)	723 (47.3)	541 (68.3)	1923 (45.6)
Oral health literacy	Oral health knowledge	10.12 ± 2.42	10.32 ± 2.45	10.05 ± 2.34	10.18 ± 2.42
	Oral health attitudes	8.41 ± 1.91	7.66 ± 2.14	7.13 ± 2.36	7.90 ± 2.14
	Oral health practices	18.70 ± 3.63	18.53 ± 3.66	18.34 ± 3.63	18.57 ± 3.64

Note: continuous variables are presented as range and mean ± standard deviation, categorical variables are presented as number (%)

### Age-stratified association between independent and outcome variables

In Table 2, oral health knowledge and pre-frailty were associated in participants aged 60–69 years (AOR = 1.05, 95% CI: 1.00–1.10) and 70–79 years (AOR = 1.08, 95% CI: 1.03–1.13). However, a significant association with frailty was seen only in participants aged 70–79 years (AOR = 1.08, 95% CI: 1.02–1.15). In all age groups, oral health attitudes were linked to both pre-frailty and frailty at all age groups (*P* < 0.05), whereas significant association of oral health practices was not observed (*P* > 0.05).

**Table 2:** Results of the logistic regression models on the association between oral health literacy and frail status among different age groups †

Variables	60–69 years				70–79 years				≥ 80 years			
	Pre-frailty		Frailty		Pre-frailty		Frailty		Pre-frailty		Frailty	
Oral health literacy	AOR	95% CI	AOR	95% CI	AOR	95% CI	AOR	95% CI	AOR	95% CI	AOR	95% CI
Oral health knowledge	1.05*	1.00–1.10	1.04	0.97–1.10	1.08**	1.03–1.13	1.08**	1.02–1.15	1.06	0.98–1.14	1.03	0.94–1.13
Oral health attitudes	0.85***	0.80–0.89	0.79***	0.74–0.85	0.88***	0.84–0.93	0.84***	0.78–0.90	0.89*	0.82–0.98	0.82***	0.74–0.91
Oral health practices	1.01	0.97–1.04	0.96	0.91–1.01	1.01	0.98–1.04	0.97	0.93–1.01	1.00	0.95–1.06	1.03	0.96–1.09

Note: Adjusted by gender, BMI, residence, living status, marital status, education, smoking and drinking status, income, depressive status, functional ability

†Reference group: non-frail group; AOR: Adjusted Odds Ratio; 95% CI: confidence interval of 95%; *P<0.05,**P<0.01,***P<0.001

### Gender-stratified association between independent and outcome variables

Table 3 presents the results of the logistic regression models after adjusting the co-variables. Oral health knowledge was correlated with pre-frailty among males (AOR = 1.09, 95% CI: 1.04–1.15) and females (AOR = 1.04, 95% CI: 1.00–1.08), but oral health knowledge was linked to frailty only in female (AOR = 1.05, 95% CI: 1.00–1.10). In both males and females, oral health attitudes were significantly correlated with pre-frailty and frailty (*P* < 0.05), whereas oral health practices were not (*P* > 0.05).

**Table 3:** Results of the logistic regression models on the association between oral health literacy and frail status among different genders†

Variables	Male				Female			
	Pre-frailty		Frailty		Pre-frailty		Frailty	
Oral health literacy	AOR	95% CI	AOR	95% CI	AOR	95% CI	AOR	95% CI
Oral health knowledge	1.09***	1.04–1.15	1.06	0.98–1.13	1.04*	1.00–1.08	1.05*	1.00–1.10
Oral health attitudes	0.86***	0.81–0.92	0.79***	0.73–0.86	0.87***	0.83–0.91	0.83***	0.78–0.87
Oral health practices	1.01	0.97–1.04	0.97	0.93–1.02	1.00	0.97–1.03	0.98	0.95–1.01

Note: Adjusted by age, BMI, residence, living status, marital status, education, smoking and drinking status, income,

depressive status, functional ability

†Reference group: non-frail group; AOR: Adjusted Odds Ratio; 95% CI: confidence interval of 95%

*P<0.05, ***P<0.001

### Residence-stratified association between independent and outcome variables

As shown in Table 4, oral health knowledge, among rural participants, was significantly linked to frailty (AOR = 1.06, 95% CI: 1.01–1.12). Among urban and rural participants, oral health attitudes were significantly related to pre-frailty and frailty (*P* < 0.05). Moreover, oral health practices were associated with frailty only among urban participants (AOR = 0.96, 95% CI: 0.92–1.00).

**Table 4:** Results of the logistic regression models on the association between oral health literacy and frail status among different residences†

Variables	Urban				Rural				
	Pre-frailty		Frailty		Pre-frailty		Frailty		
Oral health literacy	AOR	95% CI	AOR	95% CI	AOR	95% CI	AOR	95% CI	
Oral health knowledge	1.07**	1.02–1.11	1.04	1.98–1.10	1.05*	1.01–1.10	1.06*	1.01–1.12	
Oral health attitudes	0.89***	0.85–0.93	0.81***	0.76–0.87	0.86***	0.81–0.90	0.81***	0.76–0.86	
Oral health practices	1.00	0.97–1.03	0.96*	0.92-1.00	1.01	0.98–1.05	0.99	0.96–1.04	

Note: Adjusted by age, gender, BMI, living status, marital status, education, smoking and drinking status, income,

depressive status, functional ability

†Reference group: non-frail group; AOR: Adjusted Odds Ratio; 95% CI: confidence interval of 95%

*P<0.05,**P<0.01,***P<0.001

### Educational attainment-stratified association between independent and outcome variables

In Table 5, oral health knowledge was significantly related to pre-frailty among all participant groups whereas oral health knowledge was significantly associated with frailty only among participants with primary school or lower education (AOR = 1.06, 95% CI: 1.01–1.11). Oral health attitudes were significantly related to both pre-frailty and frailty among different educational attainment groups (*P* < 0.05), whereas oral health practices were not (*P* > 0.05).

**Table 5:** Results of the logistic regression models on the association between oral health literacy and frail status among different educational attainments†

Variables	Primary school and below				Junior school				High school and above			
	Pre-frailty		Frailty		Pre-frailty		Frailty		Pre-frailty		Frailty	
Oral health literacy	AOR	95% CI	AOR	95% CI	AOR	95% CI	AOR	95% CI	AOR	95% CI	AOR	95% CI
Oral health knowledge	1.05*	1.01–1.09	1.06*	1.01–1.11	1.08*	1.02–1.15	1.02	0.92–1.12	1.09*	1.00-1.18	1.06	0.94–1.19
Oral health attitudes	0.86***	0.82–0.90	0.82***	0.78–0.86	0.88***	0.81–0.95	0.84**	0.74–0.94	0.90*	0.82–0.99	0.76***	0.67–0.87
Oral health practices	1.02	0.99–1.05	0.99	0.96–1.02	0.97	0.93–1.02	0.94	0.88–1.01	1.01	0.95–1.06	1.00	0.92–1.08

Note: Adjusted by age, gender, residence, BMI, living status, marital status, smoking and drinking status, income, depressive status, functional ability

†Reference group: non-frail group; AOR: Adjusted Odds Ratio; 95% CI: confidence interval of 95%; *P<0.05,**P<0.01,***P<0.001

## Discussion

To our knowledge, this study is the first to explore the association of oral health knowledge, attitudes, and practices with frail status and whether this association is varied by age, gender, residence and educational attainment among community-dwelling older people. Based on the results, age, gender and educational attainment differences existed in the association of oral health knowledge with frail status. Urban-rural differences in the association of oral health knowledge and practices with frail status were also found.

Specifically, age differences were observed in the significant association of oral health knowledge with frailty and pre-frailty among those aged 60–69 and 70–79 years. Our findings are in line with previous research which suggested that poor oral health knowledge is more likely to be related to oral disease, which may affect the quality of life in older adults and thereby increase the potential risk of frailty [33, 34]. Similarly, the result of this paper also further illustrates the importance of oral health knowledge in maintaining the quality of life-related to oral health [35]. These results can be explained by studies that have proven that knowledge literacy is a prerequisite for cultivating healthy behaviour [36]. The poor oral knowledge reserve of Chinese older people makes it difficult to achieve behavioural changes, which indirectly affects their oral health status [37].

Our study found that gender differences in the association of oral health knowledge with frailty, while oral health attitudes and oral health practices were not. In particular, oral health knowledge was statistically significant for frailty in older females, this result may be attributed to gender differences in oral health knowledge and frailty [38, 39]. According to a prior study, females had less access to education and lacked formal or informal oral knowledge, resulting in lower levels of oral health knowledge, which led to incorrect lifestyles and affected oral health [40]. Besides, this might be explained by frailty is more common among older females [19]. Therefore, it is particularly important to prevent and control frailty by improving oral health knowledge among older females. However, in previous studies, gender differences existed in oral health attitudes and practices [21]. Compared to males, females generally paid more attention to the impact of oral health on quality of life and well-being [34]. Thus, females place more emphasis on oral health and prevention, and they have more positive attitudes toward dental visits. In addition, females are more interested in appearance and beauty, and as a result, they are more interested in flossing and brushing than males [22]. Hence, further research is needed to confirm these differences.

Meanwhile, it is worth noting that residential disparities were identified in the linkage of oral health knowledge and practices with frailty in this paper. Specifically, we determined that the correlation of oral health knowledge with frailty was statistically significant for rural, but not urban participants. This is in accordance with previous findings that older residents in rural areas generally have lower levels of oral health knowledge and poorer oral health, reducing their quality of life [23, 41] and leading to a higher risk of frailty [18]. This may also be due to the fact that rural older people have more negative oral health knowledge compared to urban older people [42]. According to a prior study which reported that older people from rural areas are unaware of the importance of regular dental cleaning and oral care services and thereby this community has a low willingness to participate and adhere to these services [43].

On the other hand, this study concluded that the association between oral health practices and frailty was statistically significant in the urban older population. Based on previous research, this result could be explained by the following reasons. First, studies have proved that urban older people have a higher consumption level of soft drinks, fruits, candies and other high-sugar products, which leads to dental problems such as cavities and tooth loss [44]. In addition, studies have shown that social participation could build up social control and improve their oral health habits [45]. In contrast, a study found that urban older people whose social network and social engagement were decreased due to infrequent social contact with their children were more likely to experience poor oral function [46]. Simultaneously, the low level of social networks of older people may also limit sources of relevant health information and reduce the utilization of dental care services [47], which are indirectly associated with frailty [48].

In contrast, the association of oral health practices with frailty was not significant after adjusting for confounders, and this was inconsistent with the results of previous studies. Studies based on the health behavior model show that changes in individual behavior would directly and indirectly lead to changes in physical and mental health outcomes [49]. For instance, a study from the Netherlands showed that oral health practices, especially lower frequency of use dental services, resulting in poorer oral health outcomes and related quality of life, are also associated with frailty-related factors in the older population [12]. Simultaneously, previous research indicated that poor oral hygiene habits (less frequent brushing of teeth and denture cleaning) lead to oral diseases and increase the risk of frailty [50]. These inconsistent results may be because different subjects from varied social backgrounds were included for analysis. Therefore, the relevant research needs to be further explored concerning these differences.

Currently, evidence from this paper has confirmed that the association of oral health knowledge with frailty had statistical discrepancies concerning educational attainments. Our study revealed that the connection between oral health knowledge and frailty was significant only in older people with primary school or lower education, which corresponds to the conclusions of a previous study [51]. Also, this may because older people with lower education levels do not fully understand or receive appropriate oral hygiene maintenance training, they may not be aware of the importance of regular dental examinations [52, 53].

Drawing on our results, the association between oral health attitudes and frail status was statistically significant, which suggested that oral health attitudes are a protective factor for pre-frailty and frailty. In other words, the more positive the oral health attitude, the lower likelihood of frailty in older people. The findings are consistent with previous research that positive attitudes accompanied by healthy behavioural habits have a direct impact on the quality of life of older people [54]. However, research from China has shown that older people living in communities often have extremely negative attitudes toward the severity of oral diseases and the maintenance of oral health [29, 55]. This may affect the perception and management of oral health, eventually affecting the maintenance of oral health, reducing the quality of life of older people, and increasing the risk of frailty [8, 29]. Therefore, improving the attitude of older people towards oral health is particularly important for intervention in frailty.

From the perspective of healthy aging, mixed-association models are committed to preventing and controlling frailty via improving oral health literacy regarding to knowledge, attitudes, and practices of older adults. At the same time, different age groups, genders, residences, and educational attainments should be fully considered to develop individualized interventions.

Nonetheless, some limitations of this study need to be pointed out. First, a cross-sectional study design may be a limitation for ascertaining a causal relationship of oral health knowledge, attitudes, and practices with frailty. Thus, longitudinal studies are warranted to further ascertain the causal relationships in future research. Second, oral health data are only obtained through self-reported measurement at the individual level, which may confer certain information biases, including recall bias and social desirability bias, etc. Despite the aforementioned limitations, we firmly believe that the use of a representative large sample and good response rates constitute the strengths of this study. The results of this study could develop our understanding of factors that would help decrease the development of frailty through good oral health knowledge, attitudes, and practices.

## Conclusion

In summary, based on the results of our study, we observed the linkages between oral health knowledge, attitudes, practices and frailty across demographic variables among community-dwelling older people. The correlation of oral health knowledge and practices with frailty varied with age, gender, residence, and educational attainments, whereas oral health attitudes were not. Our results cast a new light on the development of individualized strategies that can be tailored to improve frailty from the perspective of oral health literacy, which are crucial for achieving healthy aging.

### Electronic supplementary material

Below is the link to the electronic supplementary material.

Supplementary Material 1

## Data Availability

The datasets analyzed during the current study are not publicly available due to data management regulations but are available from the corresponding author on reasonable request.

## Abbreviations

95%CI: Confidence interval of 95%
AOR: Adjusted Odds Ratio
BMI: Body mass index
