# Supplementary material for: Differences in the association of oral health knowledge, attitudes, and practices with frailty among community-dwelling older people in China
Source: BMC Oral Health. 2023 Oct 24;23:782. doi: 10.1186/s12903-023-03477-y (PMC10594714; doi:10.1186/s12903-023-03477-y)
Supplement: Supplementary file 1 — Supplementary Material 1 [file 12903_2023_3477_MOESM1_ESM.docx]

**Frailty assessment Scale**

**1. Body Frailty**

**1.1** Do you feel that your health is significantly worse than last year?

① Obvious ② Less obvious ③ Not obvious

**1.2** Have you been eating a lot less recently?

① Obvious ② Less obvious ③ Not obvious

**1.3** Have you lost any sudden, noticeable weight recently? (“Significant weight loss” means a weight loss of at least 6kg in the last six months or at least 3 kg in the last one month)

① Obvious ② Less obvious ③ Not obvious

**1.4** Do you have difficulty moving your limbs because of the inconvenience in your daily life?

① Yes ② Occasionally ③ No

**1.5** Do you have difficulty maintaining your balance in your daily life?

① Yes ② Occasionally ③ No

**1.6** Do you have a language barrier that makes your daily life difficult?

① Yes ② Occasionally ③ No

**1.7** Do you have physical fatigue that makes your daily life difficult?

① Yes ② Occasionally ③ No

**1.8** Do you often feel tightness in your chest?

① Yes ② Occasionally ③ No

**1.9** Do you often feel physical pain? (pain in the limbs, head, neck, back, muscles, etc.)

① Yes ② Occasionally ③ No

**2. Mental Frailty**

**2.1** Have you been feeling down (unhappy) in the last month?

① Yes ② Occasionally ③ No

**2.2** Do you have physical fatigue that makes your daily life difficult?

① Yes ② Occasionally ③ No

**2.3** Have you been able to deal with the problems in your life in the last month?

① Able ② Can sometimes ③ Unable

**2.4** Have you been able to enlighten yourself on the problems you have encountered in the last month?

① Able ② Can sometimes ③ Unable

**2.5** Overall, are you satisfied with your current life or job?

① Satisfaction ② Partial satisfaction ③ Dissatisfaction

**3. Social Frailty**

**3.1** Do you live alone?

① Yes ② No

**3.2** Do you often invite people to your home? (including friends, relatives, neighbors, etc.)

① Yes ② Sometime ③ No

**3.3** Do you often visit other people's homes? (including friends, relatives, neighbors, etc.)

① Yes ② Sometime ③ No

**3.4** Do you think there are a lot of friends and relatives you can meet and confide in at any time? (including children, relatives, neighbors and friends)

① More ② Sometime ③ No

**3.5** Do you often feel lonely?

① Yes ② Sometime ③ No

**4. Environmental Frailty**

**4.1** Do you think your house is in bad condition?

① Bad ② Not too bad ③ Not bad

**4.2** Do you think your accommodation is not comfortable enough?

① Uncomfortable ② Not feel well ③ Comfortable

**4.3** Don't you like the surroundings of the house?

① Dislike ② Not too like ③ Like

**4.4** Do you think the transportation around your house is not easy?

① Yes ② Sometime ③ No

**Oral health knowledge, attitudes, and practices Questionnaire**

**1.** **Oral health knowledge**

**1.1** Are you correct in your opinion that bleeding from brushing teeth is normal?

① Correct ② Unknown ③ Wrong

**1.2** Is it true that bacteria can cause gum inflammation?

① Correct ② Unknown ③ Wrong

**1.3** Is it true that brushing teeth does not help prevent bleeding gums?

① Correct ② Unknown ③ Wrong

**1.4** Is it true that bacteria can cause dental caries?

① Correct ② Unknown ③ Wrong

**1.5** Is it true that eating sugar can cause dental caries?

① Correct ② Unknown ③ Wrong

**1.6** Are you correct that fluoride has no effect on protecting teeth?

① Correct ② Unknown ③ Wrong

**1.7** Are you correct in your opinion that fossa closure is not useful for tooth protection?

① Correct ② Unknown ③ Wrong

**1.8** Do you think that oral diseases may affect the overall health of the statement is correct?

① Correct ② Unknown ③ Wrong

**2.** **Oral health attitudes**

**2.1** You have a good opinion of your overall health.

① Agree ② Partially Disagree ③ Disagree

**2.2** You have a good opinion of your oral health.

① Agree ② Partially Disagree ③ Disagree

**2.3** Your opinion on the statement that oral health is very important to your life.

① Agree ② Partially Disagree ③ Disagree

**2.4** What do you think of the statement that regular oral examination is necessary.

① Agree ② Partially Disagree ③ Disagree

**2.5** How do you feel about the statement that good or bad teeth are innate and have little to do with your own protection.

① Agree ② Partially Disagree ③ Disagree

**2.6** Your opinion on the saying that the prevention of dental disease should first depend on oneself.

① Agree ② Partially Disagree ③ Disagree

**3.** **Oral health practices**

**3.1** Number of teeth brushing per day.

① More than 2 times ② 2 times ③ 1 time ④ 0 times

**3.2** Number of teeth brushing with toothpaste.

① More than 2 times ② 2 times ③ 1 time ④ 0 times

**3.3** Number of teeth brushing with fluoride toothpaste.

① More than 2 times ② 2 times ③ 1 time ④ 0 times

**3.4** Number of flossing.

① Daily ② Weekly ③ Occasional ④ No use/Unknown

**3.5** Use of toothpicks.

① Daily ② Weekly ③ Occasional ④ No use/ Unknown

**3.6** Frequency of eating sweet Dim sum.

① Never/Few or 1-3 times a month ② Once a week ③ 2-6 times a week ④ More than once a day

**3.7** Frequency of sweet drinks.

① Never/Few or 1-3 times a month ② Once a week ③ 2-6 times a week ④ More than once a day

**3.8** Frequency of drinking sweetened milk/yogurt/milk powder/tea/soybean milk/coffee.

① Never/Few or 1-3 times a month ② Once a week ③ 2-6 times a week ④ More than once a day

**3.9** Frequency of smoking.

① Never/Few or 1-3 times a month ② Once a week ③ 2-6 times a week ④ More than once a day

**3.10** Frequency of drinking.

① Never/Few or 1-3 times a month ② Once a week ③ 2-6 times a week ④ More than once a day

**3.11** Frequency of taking the initiative to go to the hospital for dental examination.

① Never/Few ② Every 4-6 months/time ③ Every 3 months/time ④ Once a month

**3.12** Toothbrush replacement frequency.

1. Never/Few ② Every 4-6 months/time ③ Every 3 months/time ④ Once a month
